# Determining the Delamanid Pharmacokinetics/Pharmacodynamics Susceptibility Breakpoint Using Monte Carlo Experiments

**DOI:** 10.1128/aac.01401-22

**Published:** 2023-03-06

**Authors:** Yongge Liu, Mischka Moodley, Jotam G. Pasipanodya, Tawanda Gumbo

**Affiliations:** a Otsuka Pharmaceutical Development & Commercialization, Inc., Rockville, Maryland, USA; b Otsuka Novel Products GmbH, Munich, Germany; c Quantitative Preclinical & Clinical Sciences Department, Praedicare Inc., Dallas, Texas, USA

**Keywords:** early bactericidal activity, hollow fiber system model of TB, mouse tuberculosis model, pharmacometrics

## Abstract

Antimicrobial susceptibility testing, based on clinical breakpoints that incorporate pharmacokinetics/pharmacodynamics (PK/PD) and clinical outcomes, is becoming a new standard in guiding individual patient therapy as well as for drug resistance surveillance. However, for most antituberculosis drugs, breakpoints are instead defined by the epidemiological cutoff values of the MIC of phenotypically wild-type strains irrespective of PK/PD or dose. In this study, we determined the PK/PD breakpoint for delamanid by estimating the probability of target attainment for the approved dose administered at 100 mg twice daily using Monte Carlo experiments. We used the PK/PD targets (0- to 24-h area under the concentration-time curve to MIC) identified in a murine chronic tuberculosis model, hollow fiber system model of tuberculosis, early bactericidal activity studies of patients with drug-susceptible tuberculosis, and population pharmacokinetics in patients with tuberculosis. At the MIC of 0.016 mg/L, determined using Middlebrook 7H11 agar, the probability of target attainment was 100% in the 10,000 simulated subjects. The probability of target attainment fell to 25%, 40%, and 68% for PK/PD targets derived from the mouse model, the hollow fiber system model of tuberculosis, and patients, respectively, at the MIC of 0.031 mg/L. This indicates that an MIC of 0.016 mg/L is the delamanid PK/PD breakpoint for delamanid at 100 mg twice daily. Our study demonstrated that it is feasible to use PK/PD approaches to define a breakpoint for an antituberculosis drug.

## INTRODUCTION

Drug susceptibility testing (DST) is critical for responsible antimicrobial drug use while ensuring the best clinical outcome for the patient. To accurately define susceptibility versus resistance of a bacterial isolate, a predefined drug concentration is needed. For tuberculosis (TB), this is usually referred to as the critical concentration, which is defined as the lowest concentration of an anti-TB agent that will inhibit the growth of 99% of phenotypically wild-type strains of Mycobacterium tuberculosis complex *in vitro*; the epidemiological cutoff value (ECOFF) encompasses 99% of wild type isolates in the Gaussian-shaped MIC distribution (1). This concentration is obtained by determining the MIC of a large collection of clinical isolates that are susceptible or resistant to the agent. A major disadvantage of the use of critical concentration in TB DST is that it does not always correlate with the likelihood of a patient responding to the antibiotic. This is because the critical concentration is independent of the dose administered to patients and thus drug concentrations achieved at the site of infection in patients; also, it does not reflect pharmacokinetic variability of drugs, which is the highest-ranked determinant of clinical outcomes to antimicrobial chemotherapy (2–9). Furthermore, a recent publication has highlighted the additional pitfalls associated with using the critical concentration for DST of anti-TB drugs with specific reference to the rifamycins (10). In contrast, DST for most other antimicrobials uses a pharmacokinetic/pharmacodynamic (PK/PD) breakpoint, which is the concentration that defines an MIC that separates strains that will likely respond to treatment from those which will likely not respond to treatment at a certain dose (11). There are a multitude of likely reasons for using the critical concentration rather than PK/PD breakpoint for anti-TB drugs. These include the difficulty of obtaining PK/PD data for drugs used in TB treatment, the long duration of anti-TB therapy in contrast to other therapy for bacterial infections, and the difficulty of accurately assessing individual drug contributions within a combination therapy.

Defining a PK/PD susceptibility breakpoint follows a set process (12). It requires (i) evaluation of efficacy in nonclinical settings as well as from clinical studies to derive PD targets required for efficacy, (ii) the population PK parameter and variance estimates of the drug, (iii) the MIC distribution in clinical isolates as encountered in the clinic, and (iv) Monte Carlo experiments (MCEs) to estimate exposures of the antimicrobial agent in the target patient population at specified doses (13, 14). The MCE probability of target attainment (PTA) is then determined across the MIC range for PK/PD exposures encountered in 10,000 subjects (13–15).

There has been a strong push from regulators, clinicians, and researchers in the TB field to embrace modern PK/PD modeling and analysis to better define the breakpoints used in the susceptibility testing of anti-TB drugs in patient management (10, 16–19). Fortunately, anti-TB drugs developed in the last decade have undergone more stringent evaluation utilizing modern pharmaceutical development principles. This provides an opportunity to determine whether the PK/PD breakpoint analysis used for other antibiotics can also be applied to anti-TB drugs during development and for patient management once approved. Here, we report the results of a population PK/PD analysis followed by MCEs to determine the breakpoints for a 100-mg twice-daily delamanid (Otsuka Pharmaceutical Co., Ltd., Tokyo, Japan) treatment regimen based on PK/PD targets (0- to 24-h area under the concentration-time curve [AUC_0–24_]/MIC) identified in a murine chronic TB model, hollow fiber system of TB (HFS-TB), and early bactericidal activity (EBA) studies in patients.

Delamanid is a nitroimidazole derivative that was approved by the European Medicines Agency in 2014 as an anti-TB drug that may be used as part of an appropriate combination regimen for pulmonary multidrug-resistant tuberculosis (MDR-TB) in adults, adolescents, children, and infants with a body weight of at least 10 kg, when an effective treatment regimen cannot otherwise be composed for reasons of resistance or tolerability. In addition, in combination with several other new agents, it is being examined for the possibility of establishing shorter-duration pan-TB regimens (20). In this study, we built on population PK and PK/PD work published in the past to identify the delamanid PK/PD breakpoint using MCEs (21, 22). The PK/PD targets in these prior studies, defined by exposure mediating 80% of maximal effect (EC_80_), were AUC_0–24_/MIC ratios of 252 in the mouse chronic TB model, 195 in HFS-TB with log-phase M. tuberculosis, 201 in the HFS-TB at pH 5.8, and 171 in clinical EBA studies (21).

## RESULTS

### MCE population PK model.

**FIG 1:**
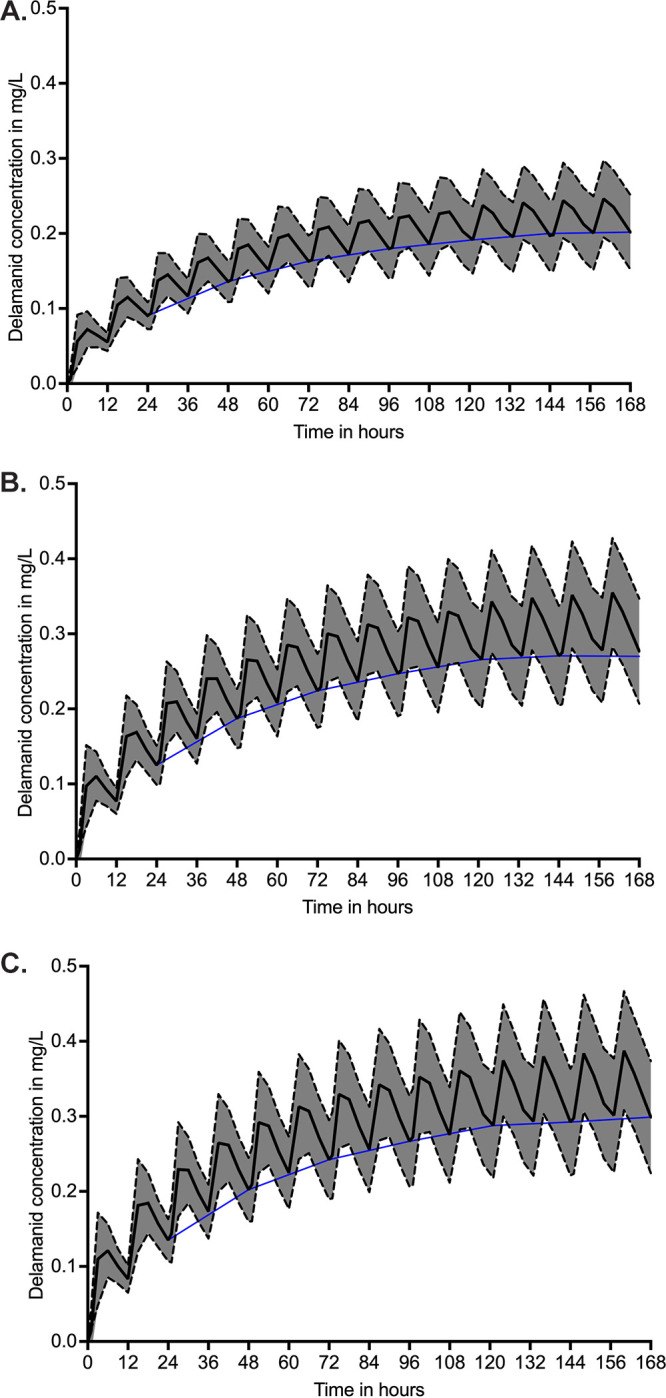
Delamanid concentrations achieved in 10,000 patients in three pharmacokinetic groups. Shown are plasma concentration-time profiles in 10,000 virtual subjects treated with delamanid 100 mg twice a day for 7 days. The black line depicts the mean concentration and the shaded area represent the standard deviations. The blue line depicts the mean trough concentrations. For all three pharmacokinetic populations, steady state was achieved by day 7. (A) Non-Asian populations (rest of the world); (B) Southeast Asian population; (C) Northeast Asian population. Concentrations on each day were lowest in the non-Asian population.

**FIG 2:**
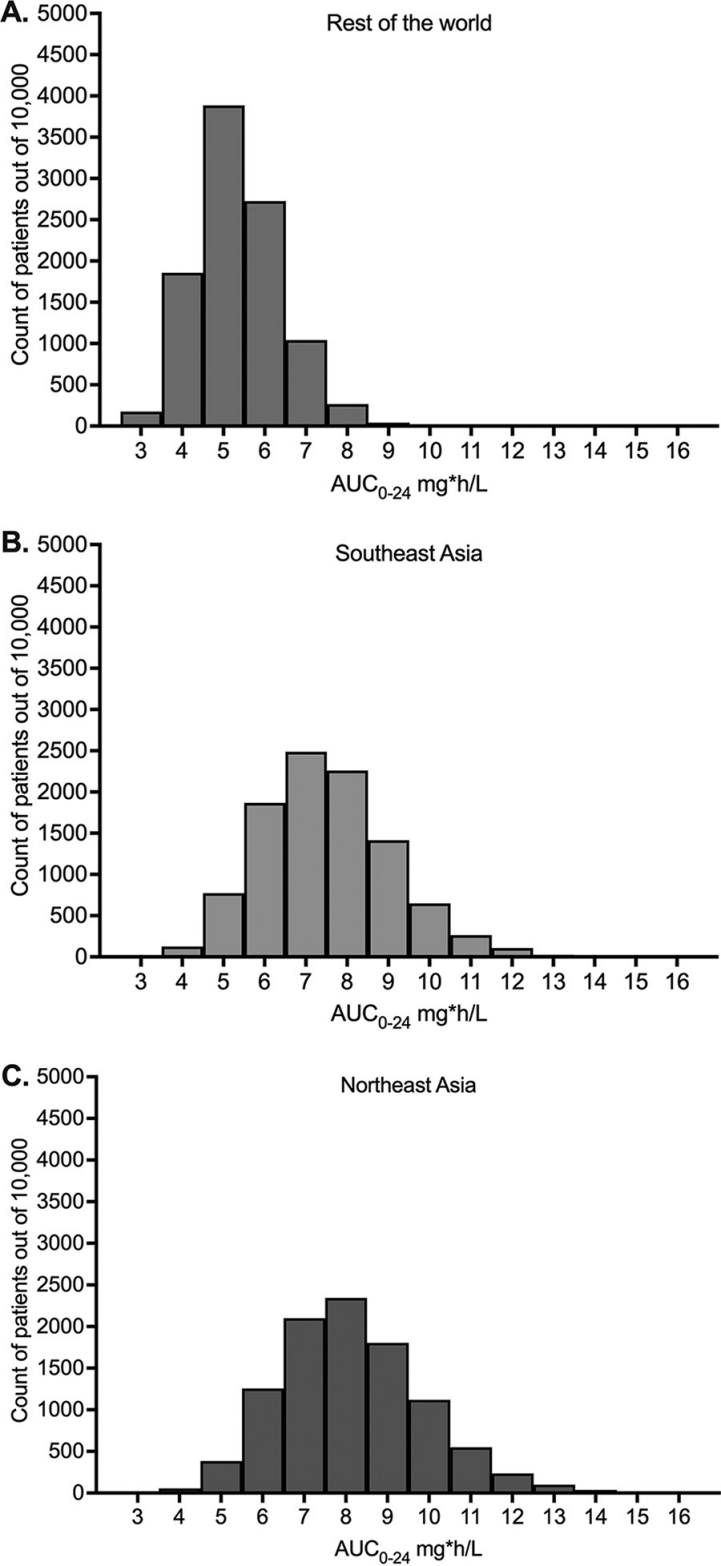
Area under the concentration-time curve distributions in 10,000 subjects. The distributions of AUCs show that the populations simulated were distinct from each other. (A) For the non-Asian populations, the steady-state median AUC_0–24_ was 5.27 mg·h/L (minimum, 2.61 mg·h/L; maximum, 10.27 mg·h/L; coefficient of variation [CV], 19.27%). The steady-state median peak concentration was 0.26 mg/L (minimum, 0.16 mg/L; maximum, 0.50 mg/L; CV, 18.71%). (B) For the Southeast Asian population, the steady-state median AUC_0–24_ was 7.38 mg·h/L (minimum, 3.26 mg·h/L; maximum, 16.63 mg·h/L; CV, 21.32%). The steady-state median peak concentration was 0.36 mg/L (minimum, 0.17 mg/L; maximum, 0.74 mg/L; CV, 20.13%). (C) For the Northeast Asian population, the steady-state median AUC_0–24_ was 7.99 mg·h/L (minimum, 3.48 mg·h/L; maximum, 15.89 mg·h/L; CV, 21.23%). The steady-state median peak concentration was 0.39 mg/L (minimum, 0.18 mg/L; maximum, 0.76 mg/L; CV, 19.97%).

We performed MCEs to identify the distribution of AUC_0–24_ values in 10,000 subjects treated with the approved delamanid dose of 100 mg administered twice daily as outpatients (22). Table 1 shows the population PK parameter estimates and variances that were entered into subroutine PRIOR of ADAPT and the population PK parameter estimates and variance output from the simulations. Parameter estimates and variances were similar to those in the domain of input, thereby passing the internal validation step. The concentration-time profiles of 10,000 subjects in the MCE over the first 7 days are presented in Fig. 1 and indicate that steady state was achieved by day 7. Figure 2 shows the distribution of AUCs in the MCE achieved in plasma of 10,000 subjects at steady state in three population PK groups (Southeast Asia [SEA], Northeast Asia [NEA], and the rest of the world). These values are similar to those measured and reported in clinical trials for delamanid given at 100 mg twice daily (22) and indicate the impact of ethnicity on final AUCs achieved.

**TABLE 1:** Population pharmacokinetic parameter estimates and variance^*a*^

Parameter	Subroutine PRIOR (*n* = 744)		Northeast Asia (*n* = 10,000)		Southeast Asia (*n* = 10,000)		Rest of world (*n* = 10,000)	
	Estimate	ω^2^	Estimate	ω^2^	Estimate	ω^2^	Estimate	ω^2^
CL (L/h)	37.1	0.06	37.3	0.06	37.1	0.06	37.3	0.06
*V*_1_ (L)	655	0.15	659	0.58	653.2	0.15	654	0.15
*Q* (L/h)	104	0.46	104	0.38	102.4	0.36	104	0.40
*V*_2_ (L)	870	0.15	871	0.15	867.1	0.15	870	0.15
*k_a_* (L/h)	0.397	0.52	0.61	0.50	0.57	0.55	0.398	0.53
Tau (h)	1.38	0.36	1.38	0.37	1.38	0.37	1.38	0.37

a CL, clearance; *V*_1_, volume of compartment 1; *Q*, intercompartmental clearance; *V*_2_, volume of compartment 2; *k_a_*, absorption rate constant; tau, lag time; ω^2^, variance on an interindividual variability term.

### Probability of target attainment of delamanid at 100 mg twice daily.

**FIG 3:**
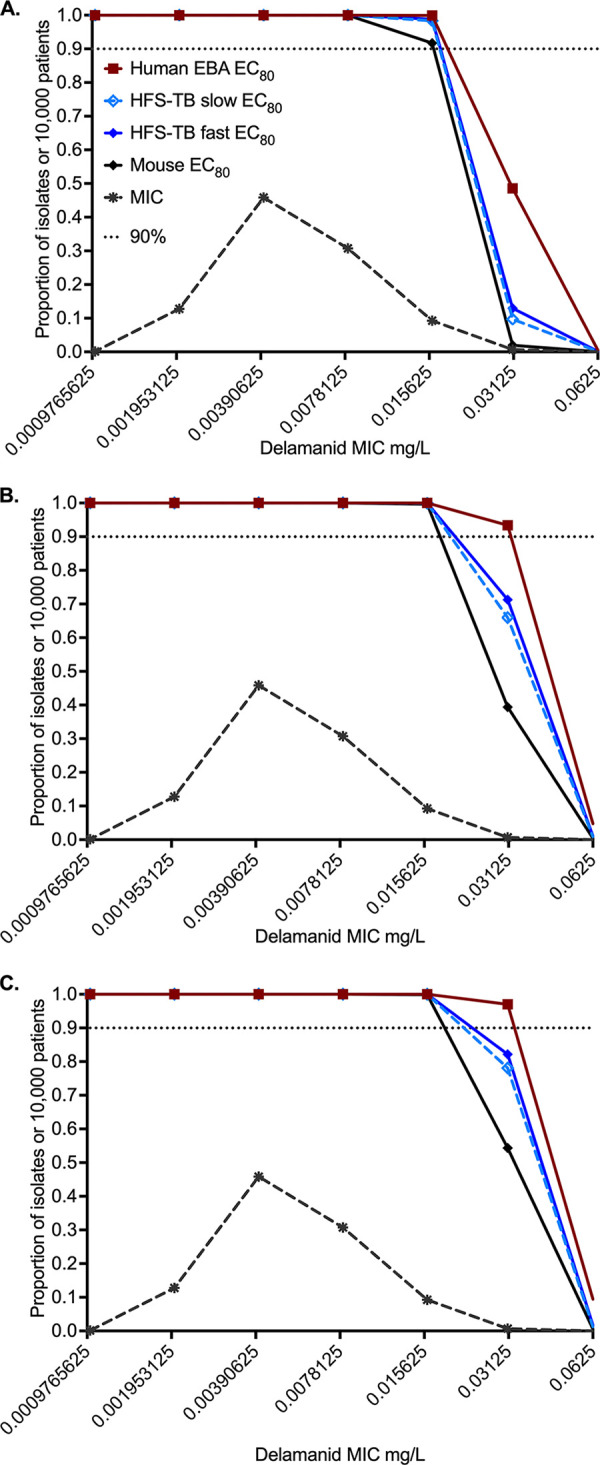
Probability of target attainment (PTA) in the different populations. PTA was taken at each MIC. The MIC distribution reflects MICs obtained from 735 Mycobacterium tuberculosis isolates obtained from patients participating in Otsuka clinical trials. (A) PTAs using population pharmacokinetic data from the rest of the world. (B) PTAs using population pharmacokinetic data from Southeast Asia. (C) PTAs using population pharmacokinetic data from Northeast Asia. Symbols represent point estimates. EBA, early bactericidal activity; EC_80_, exposure mediating 80% of maximal effect; HFS-TB, hollow fiber system model of tuberculosis.

Since the AUC distribution profiles are different among the patient groups from SEA, NEA, and the rest of the world, PTAs at various MICs and PK/PD targets were calculated based on each population, as shown in Table 2 and Fig. 3. The MIC distribution in Fig. 3 was from 735 clinical isolates. At an MIC of 0.016 mg/L or lower, the PTA was above 90% regardless of the PK/PD target used in the calculation for the three different patient populations. This was also true when the PTA was calculated using a global population PK profile based on world TB case mix calculated using the populations sizes and the incidence rates of TB in 2019 as reported by the WHO: for SEA (Philippines and other countries in that region) at 6%, NEA for the Indo-China region as far north as Japan at 35%, and the rest of the world at 59% (23); PTA results are shown in Table 3. Finally, the estimated cumulative fraction of response for all three populations was above 98% at the delamanid dose of 100 mg administered twice a day, regardless of subject population (NEA or SEA or rest of the world) or PK/PD target (HFS-TB versus murine versus clinical EBA study). This demonstrates that the PK/PD approach is robust.

**TABLE 2:** Probability of target attainment for different PK/PD targets

Pharmacokinetic group	MIC (mg/L)	PTA based on different studies			
		Human EBA	HFS-TB (pH 5.8)	HFS-TB (log-phase growth)	Mice
Rest of the world	0.0005	1	1	1	1
	0.001	0.9998	0.9998	0.9998	0.9998
	0.002	0.9998	0.9998	0.9998	0.9998
	0.004	0.9998	0.9998	0.9998	0.9998
	0.006	0.9998	0.9998	0.9998	0.9998
	0.008	0.9998	0.9998	0.9998	0.9998
	0.016	0.9991	0.9835	0.9881	0.9168
	0.032	0.4854	0.0969	0.1294	0.0196
	0.064	0	0	0	0

Southeast Asia	0.0005	1	1	1	1
	0.001	1	1	1	1
	0.002	1	1	1	1
	0.004	1	1	1	1
	0.006	1	1	1	1
	0.008	1	1	1	1
	0.016	1	0.9995	0.9999	0.9964
	0.032	0.9337	0.6598	0.7129	0.394
	0.064	0.0385	0.0022	0.0034	0.0002

Northeast Asia	0.0005	1	1	1	1
	0.001	1	1	1	1
	0.002	1	1	1	1
	0.004	1	1	1	1
	0.006	1	1	1	1
	0.008	1	1	1	1
	0.016	1	0.9999	1	0.9988
	0.032	0.9701	0.781	0.8217	0.5435
	0.064	0.0855	0.0048	0.0086	0.0002

**TABLE 3:** Probability of target attainment for 10,000 patients reflecting world TB case mix

MIC (mg/L)	Probability of target attainment (%)			
	Human early bactericidal activity	HFS-TB (pH 5.8)	HFS-TB (log-phase growth)	Mice
0.064	3	0	1	0
0.032	68	39	40	25
0.016	100	100	100	100
0.008	100	100	100	100

## DISCUSSION

Although PK/PD analyses to determine PK/PD breakpoints have been performed for antimicrobial agents for decades (24–26), these have not been commonly performed for anti-TB drugs, likely due to the difficulties of gathering relevant data. Anti-TB DST is mainly based on a critical concentration derived from ECOFFs (10, 13, 14, 27). However, a review of first- and second-line TB drugs identified that the ECOFF breakpoints may not reflect the clinical isolates response to therapy (10). Indeed, when PK/PD-based approaches were retrospectively applied to the older TB drugs, they were found to be very different from ECOFF breakpoints that were being used in the clinic, and not surprisingly, the ECOFF breakpoints did not correlate with patient responses while the PK/PD-derived breakpoints did (28–35). In recent years, the WHO World TB Programme commissioned a system of reports based on systemic reviews for (i) critical concentrations of drugs used to treat MDR-TB, including delamanid, and (ii) drug PK/PD in treatment of TB that included HFS-TB-based EC_80_-derived susceptibility breakpoints and a special one on critical concentrations of rifamycins and isoniazid, as summarized by recent reports (10, 27, 36, 37). The approach led to the lowering of critical concentrations of rifampin (which will be revisited when higher doses are used), amikacin, levofloxacin, and moxifloxacin and the introduction of a “susceptible-dose dependent” category (10). The cycloserine breakpoint hitherto used was withdrawn and has since been reidentified based on HFS-TB EC_80_ and MCE (10, 38, 39). Therefore, according to WHO reports, “fundamentally, MIC, PK/PD and clinical data should be fully integrated when setting breakpoints” (see page 5 of reference 10). It has been shown in non-TB antimicrobial work that breakpoints derived using the ECOFF and other deterministic approaches may actually amplify antimicrobial resistance, leading to clinical failure; in TB patient management, these non-PK/PD approaches could contribute to acquired TB drug resistance via the antibiotic resistance arrow of time (7, 24, 32, 40). In the antibiotic resistance arrow-of-time model, suboptimal anti-TB drug concentrations relative to the MIC initiates a specific sequence of steps comprising early induction of reversible low-level resistance which facilitates eventual emergence of chromosomal mutations associated with high-level resistance (7, 32, 40).

In this study, we applied the approaches that are commonly part of early clinical development for non-TB antimicrobials to conduct a PK/PD analysis for delamanid using two orthogonal laboratory preclinical models (murine and HFS-TB) and early clinical trial data. We were able to demonstrate that the same approaches can be prospectively applied to anti-TB drug development. We previously found that PK/PD targets identified in the mouse chronic and HFS-TB models were similar to those from patient data for delamanid (21), indicating the value of preclinical data in the determination of susceptibility breakpoints of drugs in TB. As far as we are aware, this is the first comprehensive attempt to prospectively define a PK/PD breakpoint for an anti-TB drug and recommend it for use in the clinic, in clinical trials, and for regulatory approval. The delamanid critical concentration breakpoint had been variously proposed as 0.2 mg/L or 0.04 mg/L or, coincidentally, 0.016 mg/L based on ECOFF (1, 41, 42). This could be a reflection of evolution driven regional variability in M. tuberculosis MIC distributions, hence 95% cutoff on the Gaussian (ECOFF), which is one of the drawbacks of ECOFF (43). For all other bacterial species, MIC distributions differ by geographic location. On the other hand, our PK/PD-derived breakpoint was an MIC of 0.016 mg/L and will be invariant to geographic location and the specifics of M. tuberculosis isolate collection.

In other bacterial diseases, a microbial kill of 1.0 or 2.0 log_10_ CFU/mL is used for target selection to identify susceptibility breakpoints (14). For TB, EC_80_ was chosen for PK/PD target selection based on five factors. First, we have shown elsewhere that kill rates in the HFS-TB (and animal models) such as 1.0 log_10_ CFU/mL in the first week are not related to kill rates in patient’s lung or sputa in a linear fashion but rather require nonlinear multistep functions for the translation (44). Thus, 1.0-log_10_ CFU/mL M. tuberculosis kill in preclinical models does not mean 1.0-log_10_ CFU/mL kill in patients with TB, and so its meaning is unclear. Second, a comprehensive analysis of all preclinical models (HFS-TB, mice, and guinea pigs) and patients demonstrated that the EC_50_ and EC_80_, based on the inhibitory sigmoid maximum-effect (*E*_max_) model, were mathematically invariant (i.e., did not change) across all these models and patients (17, 45). Third, a systematic analysis comparing HFS-TB-derived and animal model-derived EC_80_s for use with MCE for target attainment demonstrated concordance of identified optimal drug exposures and doses with clinical responses in patients with TB (46). Fourth, in a formal analysis, when HFS-TB-based EC_80_ plus MCE in forecasting PK/PD targets for (i) doses, (ii) optimal exposures, and (iii) susceptibility breakpoints that were published prior to clinical findings were compared to clinical outcomes and thresholds from agnostic artificial intelligence algorithms in 20 clinical studies of combination therapy reported after the HFS-TB, the forecasting accuracy was within 94% of the clinical value (47). As an example, a breakpoint MIC for drug A of 94 mg/L identified in HFS-TB EC_80_-derived MCEs was identified as 100 mg/L for patients who failed combination therapy in machine learning approaches that did not prespecify the threshold. Fifth, a randomized clinical trial published in 2022 (48) for linezolid dose selection (which sets up susceptibility breakpoint) and dose schedule in combination therapy identified exactly what had been predicted by the HFS-TB-derived EC_80_ and MCE 6 years earlier (49).

Our current work was made possible by four important lines of work. First, full dose-response and dose fractionation studies were carried out in the murine chronic TB model and in two types of HFS-TB to determine PK/PD driver and PD targets. In particular, HFS-TB has been shown to be highly predictive of PK/PD outcomes in patients, forecasting values with 94% of those observed in the clinic, and has undergone regulatory qualification as a drug development tool (17, 46, 47, 50–54). Moreover, HFS-TB has the advantage of repetitive sampling from the systems in a manner similar to serial collection of patient sputa in clinical trials, which has allowed mathematical mapping of kill rates of different M. tuberculosis metabolic groups from HFS-TB to patients (hence EBAs, sterilizing effect), time to cure, and other time-to-event PK/PD outcomes (44, 55, 56). Second, data from the delamanid EBA trials was crucial to obtain clinical PK/PD data under monotherapy. Third, we had the advantage of precise delamanid population PK parameter estimates from a large-population PK study. Finally, MICs were obtained from 735 isolates that originated from different geographic regions, including South Africa, Peru, Estonia, Japan, and Philippines, which ensured that a wider phylogenetic representation of M. tuberculosis strains was included in the MIC distribution, allowing better generalization. These advantages mean that the susceptibility breakpoints established are expected to be robust and generalizable across the world. These four lines of work provide a framework of a minimum required data set to make identification of PK/PD-derived susceptibility breakpoints feasible in development of new TB drugs.

Finally, it is important to take the lung concentrations as well as unbound drug ratios into consideration in MCEs (9). We and others have shown that drug concentrations inside lesions are determinative of clinical and microbial outcomes in patients with TB, consistent with Paul Ehrlich’s “*Corpora non agunt nisi fixate*” concept: “antibiotics only work when they are bound to target” inside the lesion (our translation) (7, 57–59). However, the drug penetration into lesions is heterogenous but can be described as a gradient based on lesion size using mathematics of dynamical sinks (7, 9). Moreover, the concentrations inside TB lung lesions are related to serum concentrations by specific ratios based on Antoine Lavoisier’s law of conservation of mass: the drug present in lung lesions comes from the blood. Thus, it is not surprising that serum drug concentrations themselves are highly predictive of patient outcomes as surrogates of lesion-based concentrations, but in a nonlinear fashion (2–6, 33, 60). In the case of delamanid, we do not have human lung lesion-based drug penetration ratios. In mice, delamanid has a total drug AUC ratio in lesions to that in plasma of 1.9 to 3; the ratio is even higher, at 18.4-fold, in the guinea pig (10, 61). We adopted the more conservative ratio of 1.9 (i.e., 190%) from murine studies as similar to human lung lesions, at the sink (lowest concentration). Delamanid protein binding is 99.5 to 99.9%, or 0.01 to 0.05% free drug; it is the free drug that is effective in microbial kill (14, 62, 63). Thus, for delamanid, the lung lesion free-drug concentrations are numerically identical to total serum concentrations of the drug.

We recognize some limitations in our susceptibility PK/PD breakpoint determination. Our results were based on bactericidal activity under monotherapy, which may not accurately reflect clinically relevant endpoints, such as culture conversion, cure, and relapse when combined with other drugs. Due to the nature of TB treatment with multiple drugs and long treatment duration, and ethical issues in conducting clinical trials in TB patients with monotherapy for longer than 14 days, PK/PD analysis using cure and relapse will be very challenging to conduct. The fact that our target exposures and PTAs were similar among mouse, HFS-TB, and human EBA studies indicates that the identified susceptibility breakpoint likely accurately reflects the antimicrobial activities of delamanid. Further, MICs depend on the assays and media used, so they would be expected to vary with different methodologies. We used the Middlebrook 7H11 agar proportion method, which is the “gold” or reference standard for MICs in TB and recommended by WHO. In addition, the methodology was standardized across the Otsuka-sponsored clinical trials to ensure that MICs were comparable (41, 64, 65). If a different methodology is used to conduct delamanid DST, then the PK/PD breakpoint may need to be calibrated from the Middlebrook 7H11 agar proportion method to the new method. This is in line with recent efforts by the European Committee on Antimicrobial Susceptibility Testing to establish a standard broth MIC test protocol and a calibration protocol and with the WHO’s technical document, which will facilitate future PK/PD analysis and PK/PD susceptibility breakpoint determination (64, 65).

In conclusion, we conducted a PK/PD analysis for delamanid and determined the PK/PD breakpoint for the 100-mg dose administered twice daily as 0.016 mg/L. Our study demonstrates the feasibility of conducting an analysis to define a PK/PD breakpoint for an anti-TB drug during drug development. This approach may be of value to refine the breakpoints used for susceptibility testing of currently available TB drugs, to improve future TB drug development and ultimately individual patient outcomes.

## MATERIALS AND METHODS

### PK/PD target selection for the Monte Carlo experiments.

The PK/PD target was the EC_80_ delamanid exposure, in either mice, the hollow fiber system model of TB (HFS-TB), or clinical full exposure-response surfaces. We utilized mean parameter estimates from the PK/PD studies. Previous studies were conducted using (i) two HFS-TB models (log-phase growth and sterilizing effect at pH 5.8) and (ii) mouse chronic TB models and demonstrated that the PK/PD index linked to delamanid microbial kill is AUC_0–24_/MIC ratio, (iii) data was also collected from clinical trial EBA patients. The EC_80_s (pharmacodynamic targets) were (i) a plasma-equivalent AUC_0–24_/MIC of 195 for log-phase M. tuberculosis, (ii) a plasma-equivalent AUC_0–24_/MIC of 201 for HFS-TB at pH 5.8, (iii) a plasma AUC_0–24_/MIC of 252 in the mouse chronic TB model, and (iv) a plasma AUC_0–24_/MIC of 171 in clinical EBA studies (21). Total drug concentrations were used. The preclinical and clinical studies were PK/PD studies with delamanid monotherapy.

### Population PK parameter estimates used for the Monte Carlo experiments.

The population PK parameter estimates and variances used were based on those from a previous study of four patient populations (22). This included 517 (69.5%) male and 227 (30.5%) female patients with self-identified racial classifications as follows: white, 117 (23.8%); black, 51 (6.9%); Northeast Asian (NEA), 301 (40.5%); Southeast Asian (SEA), 200; and others, 215 (28.9%) (22). The most important covariates were ethnic groups (NEA, SEA, and the rest of the world), with the most important impact on variability of oral absorption parameters (22). The population PK parameter estimates and variance, for a two-compartment model with first-order input and elimination, and lag time were entered into subroutine PRIOR of the Fortran model (2LAGCL) file of ADAPT 5; subroutine PRIOR is the domain of input (66). The population PK parameter estimates and the variance used are shown in Table 1 and discussed in detail in Results.

### MICs used in the Monte Carlo experiments.

Delamanid MICs, using the 7H11 Middlebrook agar proportion method, were obtained from 735 baseline M. tuberculosis isolates from participants with drug-susceptible TB and MDR-TB who have participated in Otsuka-sponsored clinical trials (trial 242-07-204 [NCT00685360] and trial 242-09-213 [NCT01424670]) (21, 22). The methods for the delamanid MIC testing, and the drug concentration ranges, have been described in detail in a prior publication (41).

### Application of Monte Carlo experiments.

Input parameters of a dose of 100 mg twice a day delivered as a bolus to the gastrointestinal tract were entered into the .dat file. Both normal and log-normal distributions were examined in the models. Simulations were for 7 days of dosing, and 998 time points in the MCE outputs were examined to determine if steady state had been achieved, and if not, testing was planned to extend to 14 days. Since there was a considerable lag of absorption, we modified both *k_a_* and bioavailability by drug absorption lag time (LAG). The *k_a_* describes the rate at which a drug enters into the system (expressed per hour), while bioavailability (*F*_1_) is the fraction or extent of availability of drug to the general circulation or site of pharmacological actions (67). Bioavailability is directly dependent on both the rate and extent of drug absorption; therefore, *F*_1_ was made to directly modify both the *k_a_* and drug concentrations achieved in the serum.

Model internal validation was performed by comparing the final MCE estimates of primary population PK parameters to those in the domain of input. Drug concentration-time profiles, peak concentrations, and AUC_0–24_ for each of 10,000 virtual subjects were then generated. We performed these tasks with PK parameters for each of the patient populations of NEA, SEA, or the rest of the world. The steady-state AUC_0–24_s achieved in the 10,000 subjects, and their distributions were then identified for each of the populations. The AUC_0–24_/MIC achieved was calculated for each of the 10,000 patients using the MIC identified in the 735 isolates (Table 3). The probability of the delamanid 100-mg twice-daily dose to achieve or exceed the target (PTA) was calculated at each MIC, for each of the three different PK groups, and for each pharmacodynamic target (mice, two HFS-TB targets, and patients). Finally, data were summated at each MIC by taking an expectation over the entire MIC distribution for each of the populations (SEA, NEA, and the rest of the world). We also examined a mixed population based on the TB case distributions in the world on PTA. An adequate PTA was defined as a PTA of >90% of the 10,000 subjects.

### Software.

Graphical and all other statistical analyses, including evaluation of NONMEM outputs, was performed using R version 3.3.0 or later for Windows (R project [http://www.r-project.org/]). For the population PK modeling results and MCE presented here, ADAPT 5 software was used (66). Computer resources included the Praedicare server (“Mambo”), which represents Fortran-based population PK modeling capabilities on graphics processing units on a virtual machine.
